# Imatinib-Functionalized Galactose Hydrogels Loaded with Nanohydroxyapatite as a Drug Delivery System for Osteosarcoma: In Vitro Studies

**DOI:** 10.1021/acsomega.3c00986

**Published:** 2023-05-09

**Authors:** Paulina Sobierajska, Benita Wiatrak, Paulina Jawien, Maciej Janeczek, Katarzyna Wiglusz, Adam Szeląg, Rafal J. Wiglusz

**Affiliations:** † Institute of Low Temperature and Structure Research, Polish Academy of Sciences, Okolna 2, Wroclaw 50-422, Poland; ‡ Department of Pharmacology, Wroclaw Medical University, Mikulicza-Radeckiego 2, Wroclaw 50-345, Poland; § Department of Biostructure and Animal Physiology, Wroclaw University of Environmental and Life Sciences, Norwida 25/27, 50-375 Wroclaw, Poland; ∥ Department of Basic Chemical Sciences, Faculty of Pharmacy, Wroclaw Medical University, Borowska 211 A, 50-566 Wroclaw, Poland

## Abstract

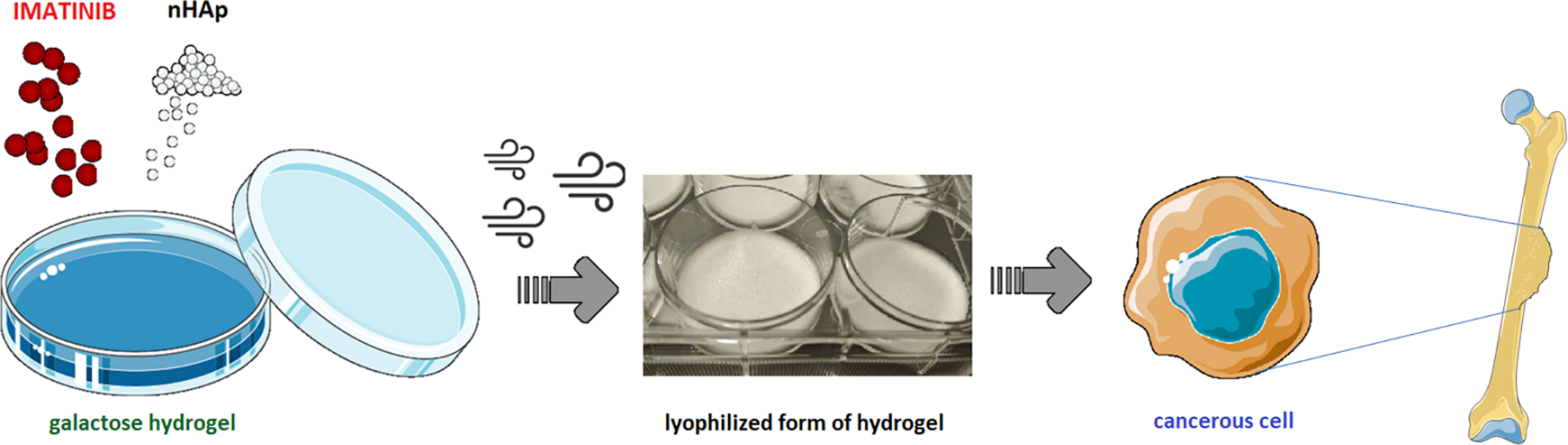

This study reports an impact of structure (XRPD, FT-IR) and surface morphology (SEM-EDS) of imatinib-functionalized galactose hydrogels, loaded and unloaded with nHAp, on osteosarcoma cell (Saos-2 and U-2OS) viability, levels of free oxygen radicals, and nitric oxide, levels of BCL-2, p53, and caspase 3 and 9, as well as glycoprotein-P activity. It was investigated how the rough surface of the crystalline hydroxyapatite-modified hydrogel affected amorphous imatinib (IM) release. The imatinib drug effect on cell cultures has been demonstrated in different forms of administration—directly to the culture or the hydrogels. Administration of IM and hydrogel composites could be expected to reduce the risk of multidrug resistance development by inhibiting P_gp_.

## 1 Introduction

Galactose derivatives, as natural polysaccharides, have a chemical structure capable of forming physical or chemical interactions with drug and bioactive molecules for delivery purposes.^1^ Agarose, alginate, cellulose, chitosan, dextrin, and starch are well-known polysaccharides widely used for drug delivery systems.^2−9^ Among them, agarose (composed of repeating units of 1,3-linked β-d-galactose and 1,4-linked 3,6-anhydro-α-l-galactose) represents reversible thermo-gelling properties, favorable mechanical properties, high bioactivity, and the ability to be functionalized for use in various medical fields.^1,10^ Moreover, unlike other polysaccharides, agarose can be dissolved in a neutral environment. Due to its neutral surface charge at different pH values, agarose can carry active substances with a low protein crown content, thus improving delivery efficiency.

Furthermore, agarose usually has low cellular attachment; hence, the researchers modified its surface to enable proper cell adhesion. Our group propose to obtain the rough structure of the agarose-based hydrogels (galactose hydrogels) by adding nanohydroxyapatite (Ca_10_(PO_4_)_6_(OH)_2_, nHAp), a bone mineral. Synthetic nHAp as a bioactive bioceramic allows osteocytes to adhere and promote osteogenesis. Thus, galactose-based hydrogels loaded with nanohydroxyapatite (nHAp) are very attractive as drug carriers due to their high biocompatibility and good cell adhesion.^11^ The strategy involving their surface functionalization might be a promising alternative to the conventional method of drug delivery, increasing the effectiveness of the therapeutic system.

Nowadays, our interest is focused on the well-described chemotherapeutic imatinib used in targeted anticancer therapies.^12^ The Saos-2 and U-2OS osteosarcoma cell lines were chosen as they are cancer cell lines derived from nanoapatite-rich bone tissue and are one of the most commonly used models for in vitro studies on the bone tissue.^13^ Moreover, Saos-2 cells reveal the most mature osteoblastic labelling profile, while the U-2OS cell line is classified not only as osteoblastic but also as fibroblastic.^14^ U2OS cells do not differentiate and do not form a calcified matrix. Saos-2 cells, on the other hand, show a high mineralization capacity, and the osteoinductive effect was determined in in vitro studies.^15^ Additionally, Saos-2 cells are p53 defective, making them much more sensitive to apoptosis than p53 expressing U-2OS cells.^16^

Osteosarcoma (OS) is an aggressive, high-grade tumor with a low survival rate (mainly in children and adolescents aged 10–20^17^) and accounts for about 60% of a malignant bone tumor.^18^ The spindle-shaped OS cells produce a cancerous osteoid (bone tissue), which is required for diagnosis.^19^ After diagnosis, multimodal therapy is most often used, and the treatment with chemotherapeutic agents (neoadjuvant and adjuvant during 6–8 months) is introduced to reduce tumor size followed by surgical removal of the neoplastic tissue and replenishment of the resection site with bone implant materials.^20,21^ Unfortunately, the effectiveness of therapy results in a 5 year survival of only 60–70% in children, while in patients with metastatic disease, the survival rate is only 10–30%.^22^ Chemotherapeutic agents with long-term anti-cancer activity in osteosarcoma include cis-platinum, doxorubicin, ifosfamide, and methotrexate.^23^ However, new, more effective drugs are being sought. One of these new generation drugs is imatinib, used in the form of imatinib mesylate (IM, Gleevec, Novartis Pharma). IM is a tyrosine kinase inhibitor (TKi, inhibits cell proliferation and enhances cell apoptosis) and was originally developed for the treatment of chronic myeloid leukemia in patients with Philadelphia chromosome (BCR-ABL, Ph+) who are ineligible for first-line bone marrow transplantation.^24^ Studies conducted in the context of OS treatment have revealed that IM inhibits osteoclast differentiation through the M-CSFR pathway and activates osteoblast differentiation through the PDGFR pathway, two key cell types involved in cancer development. Moreover, the drug causes cell death and strongly inhibits the migration of osteosarcoma cells.^25^ When administered orally, IM has been shown to significantly inhibit OS tumor growth in both a preventive and therapeutic approach.^26^ Therefore, it would be interesting to re-examine the therapeutic efficacy of imatinib by its direct administration to the target site using galactose-based biomaterials in combination with bioactive bone nanobioceramic. Nanoscale hydroxyapatite (with larger surface areas) affects biological responses, enhancing adhesion of osteoblasts and improving bone remodeling compared to non-nanophasic ceramics.^27^ Therefore, this study aims to investigate (in vitro) the influence of nHAp on the therapeutic efficacy of a drug released from the nHAp-modified IM/galactose hydrogel in comparison to the unloaded IM/galactose hydrogel.

## 2 Methodology

### 2.1 Synthesis of Nanohydoxyapatite

Ca(NO_3_)_2_·4H_2_O (≥99% Acros Organics, Schwerte, Germany), (NH_4_)_2_HPO_4_ (≥98% Avantor Performance Materials, Gliwice, Poland) and NH_3_·H_2_O (99% Avantor Performance Materials, Gliwice, Poland) were used as reagents for the preparation of nanohydroxyapatite (nHAp). First, the MQ-water solutions of calcium nitrate tetrahydrate and diammonium hydrogen phosphate were mixed and the pH was adjusted to 10 by the addition of ammonia. The obtained white precipitate (suspended in water) was stirred at 90 °C during 90 min. Then, the precipitate was centrifuged, washed with MQ-water until neutral pH, dried at 70 °C to a powder, and then thermally treated at 500 °C for 3 h.

### 2.2 Preparation of Galactose Hydrogels

Pure galactose hydrogel as well as galactose hydrogels loaded with nanohydroxyapatite (nHAp) and/or imatinib (IM) was fabricated using the freeze drying process. At first, 3,6-anhydro-α-l-galacto-β-d-galactan (Prona Agarose, BASICA LE GQT, Burgos, Spain) was dissolved in MQ-water at 70 °C and stirred for 1 h, and then when cooling down, nHAp and/or IM were added. After that, glycerine (glycerine anhydrous 99.5%, Avantor Performance Materials, Gliwice, Poland) was added. The suspension was transferred to the cell-culture plates. All obtained hydrogels were frozen and transferred to the lyophilizer to prepare a sponge-like form.

### 2.3 Characterization of Obtained Materials

The structure of the obtained nanohydroxyapatite and lyophilized hydrogels were determined using the X-ray powder diffraction (XRPD) technique with a PANalytical X’Pert Pro diffractometer (Ni-filtered Cu Kα radiation, *V* = 40 kV, *I* = 30 mA). The Fourier Transform infrared spectra (FT-IR) were detected in KBr pellets at room temperature using a Thermo Scientific Nicolet iS50 FT-IR spectrometer. The morphology of the obtained materials and elemental analysis together with the mapping of elements were done using a scanning electron microscope (SEM) FEI Nova NanoSEM 230 equipped with an energy dispersive X-ray spectrometer (EDS; EDAX PegasusXM4).

### 2.4 Imatinib Release

The release profiles of the imatinib from the hydrogels were established in PBS (phosphate-buffered saline) at 37 °C and 100 rpm rotation speed. The samples were collected at the time intervals of 0, 5, 10, and 30 min, as well as 1, 2, 6, and 24 h. The concentration of each sample was determined with ultraviolet–visible (UV–vis) measurements. The absorption spectra in the range of 230 to 450 nm (43,478–22,222 cm^–1^) were recorded on the Agilent Cary 5000 UV–vis–NIR spectrophotometer, Version 2.24 (Agilent Technologies, Santa Clara, CA, USA) with a data interval of 0.250 nm, scan rate of 150 nm/min, and spectral bandwidth of 0.500 nm. The calibration curve (λ_m_ = 263.5 nm) was prepared with the use of various concentration solutions (0 to 50 μg/mL) of the imatinib at room temperature in PBS.

### 2.5 Cell Lines and Culture Media

All bioassays were performed using two cell lines derived from osteosarcoma patients. Both U-2OS and Saos-2 were purchased from the American Type Culture Collection (ATCC). Cells were cultured in ATCC recommended medium. Cells were incubated in McCoy’s 5A medium supplemented with 10% fetal bovine serum (FBS), 2 mM l-glutamine, and 0.1 mg/mL gentamicin.

### 2.6 Cell Viability

The cytotoxicity assessment of the tested gels was performed according to ISO 10993-5: 2009. The MTT assay was performed in direct contact. Saos-2 and U-2OS cells were seeded into 96-well plates at a concentration of 10,000 cells per well. The cell cultures were then incubated overnight at 37 °C, 5% CO_2_, and 95% humidity to allow the cells to adhere. The following day, the supernatant was changed to medium with FBS reduced to 5% and then 5 × 5 mm test gels were added. Additionally, concentrations of imatinib (1, 50, 200, and 500 μM) were added as a positive control for cell culture. After 24 h, the cell cultures were washed and then 10 mg/mL MTT ((3- (4,5-dimethylthiazol-2-yl)-2) dissolved in PBS was added for 12 h of incubation under the same conditions. The supernatant was then removed, and isopropanol was added to dissolve the purple crystals. The culture plates were placed on a shaker for 30 min, after which time the absorbance was measured using a Thermo Scientific Multiscan GO microplate reader.

### 2.7 Reactive Oxygen Species (ROS) and Nitric Oxide (NO) Levels

The DCF-DA (2′,7′-dichlorofluorescin diacetate) and Griess reagent assay for oxygen-free radicals and nitric oxide levels, respectively, were used to assess the in vitro pro-oxidative activity of the tested gels and imatinib. Cell cultures were seeded into 96-well plates at a density of 20,000 cells per well. The next day, the supernatant was replaced with MEM without phenol red and FBS. Tested gels measuring 5 × 5 mm were placed on the cells. Imatinib in MEM without phenol red and FBS was added to control wells at concentrations of 1, 50, 200, and 500 μM. The cell cultures were incubated for 1 h. Then, 50 μM of the solution was transferred to new plates. The remaining solution and gels were removed, and 25 μM DCF-DA solution was added to the cultures, which were incubated in a CO_2_ incubator for another hour. To the collected solutions, mixtures of reagent A and reagent B were added at a ratio of 1: 1 *v*/*v*. The plates were incubated for 30 min in the dark. The absorbance at 548 nm was then measured using a plate reader. On the other hand, after 1 h of incubation of the cell cultures, the fluorescence was read using a Fluoroskan Ascent FL λ_ex_ = 485 nm and λ_em_ = 538 nm fluorescence reader.

### 2.8 BCL-2, P53, and Caspase 3 and 9 Levels

Invitrogen ELISA kits were used to assess the levels of p53 protein, BCL-2 protein, and caspase 3 and 9 with catalog numbers: BMS256, EHCXCL13, KH01091, and BMS2025. After 24 h incubation of the cell cultures with the tested gels and imatinib, the supernatant was collected into freezing tubes. Cells were scraped and then centrifuged for 5 min at 1000 × *g*. The cells were then resuspended in PBS and homogenized using an ultrasonic homogenizer. The cell cultures were centrifuged again, and the supernatant was collected and stored in freezing tubes. Homogenates and supernatant were stored at −80 °C. Within 1 month of sample preparation, ELISAs were performed according to the instructions provided by the manufacturer. Supernatant collected from cell culture was used to assess p53 levels, and cell homogenates were used otherwise.

### 2.9 Statistical Analysis

All bioassays were performed in triplicate. The data obtained were characterized by normal distribution and equality of variance (confirmed by Shapiro–Wilk and Levene’s tests, respectively). Therefore, statistical analysis was performed using parametric tests—one-way ANOVA and Scheffe post hoc. The point of significance was taken at *p* < 0.05. Data were presented as mean + SD for the p53, BCL-2 level, and caspases tested. In contrast, assays for cytotoxicity and ROS and NO levels were presented as an *E*/*E*_0_ ratio. *E* is the mean for a given sample, and *E*_0_ is the culture of cells only in medium without tested gels and imatinib.

## 3 Results and Discussion

**Figure 1:**
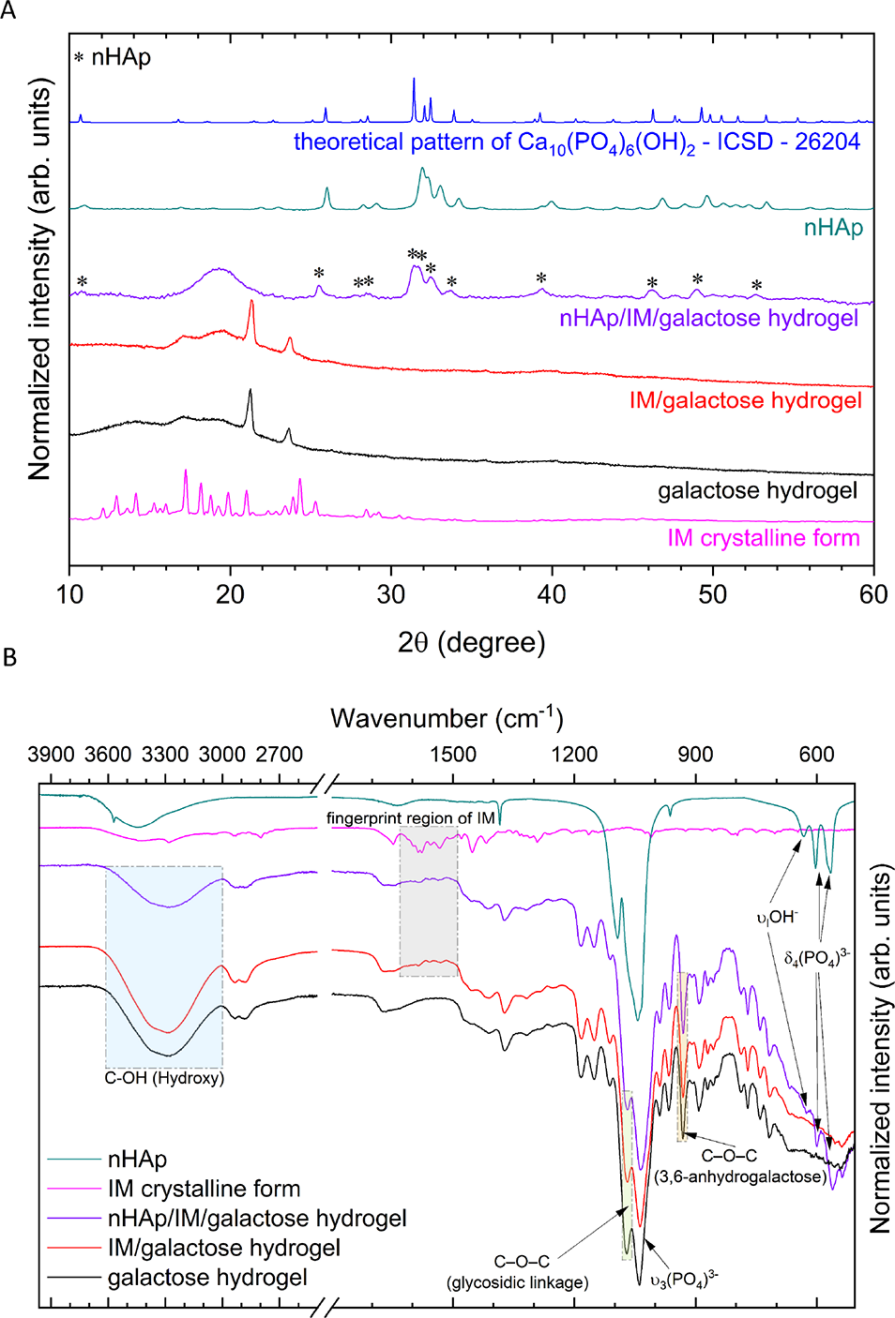
XRPD patterns (A) and FT-IR spectra (gray space, characteristic peaks for IM) (B) of the nHAp (nanopowder), imatinib (IM crystalline form), and nHAp and/or IM dispersed in the lyophilized galactose hydrogels compared to pure galactose hydrogel (blue, green, and orange spaces: characteristic peaks for the galactose hydrogel).

The X-ray powder diffraction (XRPD) technique was used to examine the structure of the obtained galactose hydrogels: nHAp/IM/galactose hydrogel and IM/galactose hydrogel. The diffraction patterns of these two hydrogels were compared to the diffractograms of separated compounds: nHAp, imatinib (IM), and galactose hydrogel (see Figure 1A). First, the IM/galactose pattern was very similar to the pattern of galactose hydrogel, indicating that imatinib had transferred from a crystalline form to an amorphous one, seen as a broad halo with a maximum at 2θ of about 20° in the XRPD pattern. This observation was also noted in our previous paper regarding the nanoapatite-mediated delivery system for imatinib.^28^ Second, in the hydrogel in which hydroxyapatite was dispersed, distinct peaks derived from nHAp were seen at 2θ equal to 25.5, 28.3, 29.1, 31.4, 32.5, 33.7, 39.3, 46.2, 49.1, and 52.6°. Moreover, all peaks observed in the nHAp/IM/galactose hydrogel and nHAp correspond to the peak position for the theoretical hexagonal hydroxyapatite (ICSD–26204^29^). These peaks are quite wide, indicating that hydroxyapatite in the nanoform was used for the preparation of hydrogels. In our previous work,^28^ the nano nature of nHAp was exhaustively described, and the mean size of the rod-like nanoparticles was 52 nm × 30 nm.

The FTIR spectra of obtained hydrogels indicate the surface interaction between nHAp, IM, and galactose matrix. It can be observed that the FT-IR spectra of the IM/galactose hydrogel (red line in Figure 1B) and nHAp/IM/galactose hydrogel (violet line, Figure 1B) are similar to that of pure IM in the fingerprint region (magenta line, Figure 1B) and in the region of *v_3_* PO_4_^3–^ vibrations of nHAp (cyan line, Figure 1B). The presence of imatinib in both types of hydrogels has been confirmed by the detection of its characteristic peaks in the range of 1490–1632 cm^–1^ (C–C and C–N stretching pyridine and aminopyrimidine ring vibrations mixed with in-plane deformation of C–H, marked as gray space in Figure 1B). The FT-IR bands of the PO_4_^3–^ groups derived from nHAp were found at 561.6, 574.0, and 599.7 cm^–1^ (the triply degenerate δ_4_ bending) as well as at 1042 and 1089 cm^–1^ (the asymmetric triply degenerate stretching *v*_3_ vibrations).^30,31^ The symmetric nondegenerate stretching *v*_1_ vibrations at about 962 cm^–1^ overlap with the bands belonging to the galactose. Moreover, the presence of the OH^–^ in the structure was confirmed by the librational vibration of OH^–^ groups at 633 cm^–1.^^32^ The typical stretching band of the C–OH group of galactose (3,6-anhydro-α-l-galacto-β-d-galactan) hydrogel is localized at the maximum position of 3296 cm^–1^ (blue space in Figure 1B). There are also visible other characteristic peaks of galactose, at 1071 cm^–1^ (vibration of C–O–C bridge of glycosidic linkage, green space, Figure 1B) and 931 cm^–1^ (vibration of C–O–C bridge of 3,6-anhydrogalactose unit, orange space, Figure 1B). The characteristic absorption band width (3200–3600 cm^–1^) for the stretching of hydrogen bonds (inter- and intra-molecular hydrogen bonds and free −OH groups^33−35^ is reduced for the nHAp/IM/galactose hydrogels. This area coincides with the N–H stretching band of IM, which is in 3350–3310 cm^–1^, and the hydrogen bonds cause N–H stretching peaks to broaden. The imatinib contains six functional groups as a potential site to hydrogen bonds (the nitrogen atoms of secondary amine and of amide) and six acceptors of H-bonding.^36^ The conclusion is that the number of free hydroxyl groups was decreased in the IM/galactose hydrogel and nHAp/IM/galactose hydrogel, and the intra- and inter-molecular hydrogen bonds have been formed between galactose hydrogel matrix and IM and/or nHAp. The significant role of the hydrogen bond can be observed by interpreting the shift of the signal from 3276 cm^–1^ for the galactose hydrogel and the IM/galactose hydrogel to 3289 cm^–1^ for the nHAp/IM/galactose hydrogel. The characteristic C=O stretching bond for secondary and tertiary amide is observed in 1648 cm^–1^ in pure form of imatinib, and the C=O band is broadened for hydrogels with IM, and it could mean that some interaction has occurred. The changes in the location of the C=O band of the drug in the polymer network indicate a modified environment caused by the formation of hydrogen bonds between the carbonyl groups of IM and the polymer. All identified bands are consistent with the literature data.^37^

**Figure 2:**
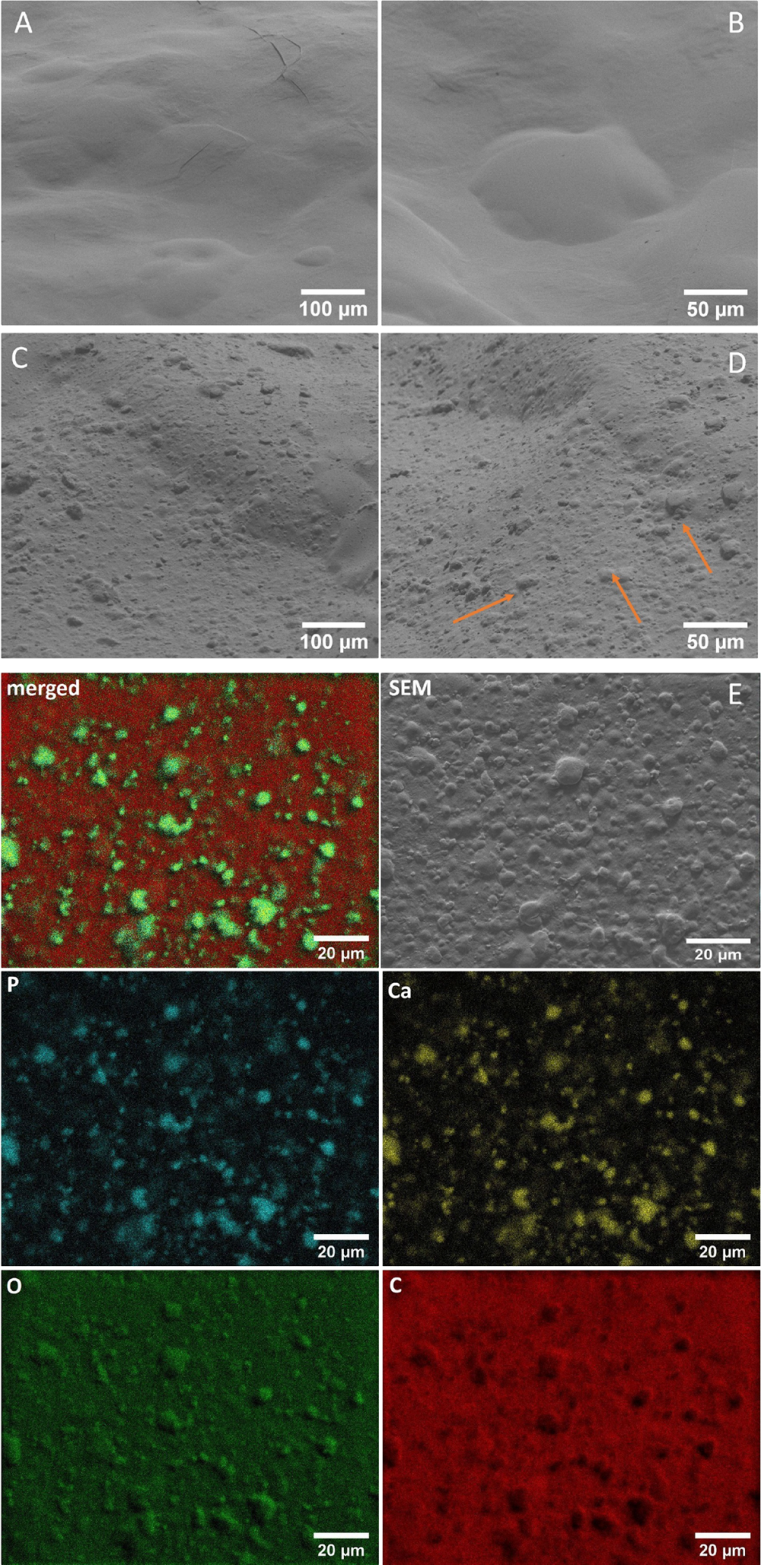
SEM images of the galactose hydrogel functionalized with imatinib (A, magnification 500×; B, magnification 1000×) as well as imatinib-functionalized galactose hydrogel loaded with nHAp (C, magnification 500×; D, magnification 1000×). Orange arrows indicate some areas with hydroxyapatite. Representative SEM-EDS elemental maps of the nHAp/IM/galactose hydrogel (E).

**Figure 3:**
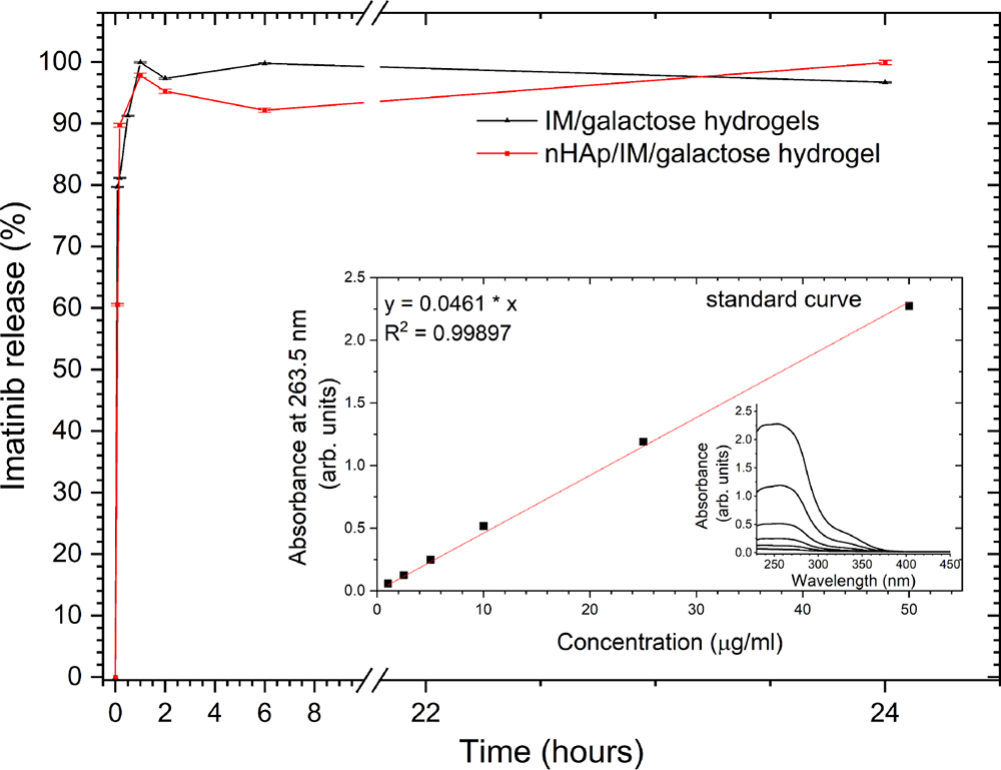
Time-dependent imatinib release from the IM/galactose hydrogel and nHAp/IM/galactose hydrogel together with the calibration curve (inset).

The SEM technique was used to observe the surface morphology galactose-based hydrogels. Figure 2A,B shows the images of the galactose hydrogel functionalized with imatinib, while Figure 2C,D presents the imatinib-functionalized galactose hydrogel loaded with nHAp. The unloaded hydrogel (without nHAp) has a rather smooth surface morphology. Incorporation of nanoapatite leads to a rough surface, with nanoparticles being clumped and covered with a hydrogel matrix (orange arrows in Figure 2D). These agglomerates are clearly visible on the elemental maps in Figure 3E. From an application point of view, a rough or porous structure is desirable—it allows better cell adhesion and cell attachment.^38^ Our previous studies confirm these conclusions.^11,39,40^

Hydrogel-mediated drug delivery systems are widely studied to improve the bioavailability of drugs and reduce adverse effects on surrounding tissues.^41,42^ Here, the release behavior of imatinib from the galactose hydrogel and galactose hydrogel modified with nHAp has been investigated. The results of the release profiles are shown in Figure 3. First, it should be noted that the release is similar in both cases. The maximum concentration (approximately 100%) of imatinib in PBS is observed after 1 h of incubation and the saturation of drug in both cases is maintained during the next 24 h. However, in the case of nHAp/IM/galactose hydrogel, the level of drug released during first few hours is slightly smaller than for the IM/galactose hydrogel. A similar observation was found in our previous research concerning time-dependent fluconazole release from the galactose hydrogels loaded and unloaded with nHAp.^40^ Moreover, the nHAp nanoparticle-mediated imatinib system was also tested previously, revealing that 100% of IM was achieved in PBS after 45 min of nHAp/IM incubation and 88% of the drug was released during the first 5 min.^28^ In this study, 60% of the drug was released after 5 min of experimental time. The gel influenced the rate of drug release, however, to a minor extent.

In patients with osteosarcoma, combination therapy is performed, comprising pharmacological treatment (chemotherapy) and surgery to remove the tumor.^43^ The primary stage is a surgical procedure of completely removing cancer with an appropriate margin of healthy tissue. Pharmacological regimens can be divided into two stages of chemotherapy: neoadjuvant and adjuvant.^44^ In the first case, the treatment is used before surgery and reduces the tumor size, thus facilitating its excision. Adjuvant chemotherapy, on the other hand, is given after tumor resection surgery. The main purpose of its use is to destroy the so-called micrometastases, which are residual tumor cells that remain in the body despite the removal of the main tumor mass.^44^ The standard oral form of cytostatic therapy shows limited high-dose potential due to toxicity to normal cells. Studies have shown that the efficacy of cisplatin, commonly used in the treatment of osteosarcoma, is limited by its nephrotoxic dose-limiting effect.^21^ Hydrogels containing nHAp and cytostatics can be used to deliver drugs to tumor sites specifically, allowing the distribution of unique effector molecules while limiting side effects.^45^ This makes it possible to apply multiple chemotherapeutics over a long period of time, which is important in the treatment of osteosarcoma and in the treatment of solid tumors with bone metastases. In these tumors, a two-drug or multidrug treatment regimen is used.^21,46^ Given the mechanisms involving different pathways in metastatic bone tumors, it is likely that combination therapy of both the bone stroma and the tumor that resides within it will be more effective than the use of a single drug.^46^ In the treatment of osteosarcoma, the most common treatment regimen is the administration of cisplatin together with doxyrubicin and high-dose methotrexate. It has also been shown that certain tyrosine kinase receptors are overexpressed in this tumor. This warrants a novel approach to metastatic treatment and the use of drugs that inhibit these receptors.^21^ The drug in this group is imatinib, which inhibits the BCR-Abl kinase, and is most commonly used in blood cancers. Unfortunately, chemotherapy administered by the oral or intravenous route in this tumor is quite limited due to the poor blood supply to the bones.^47^ This causes a significant reduction in drug delivery to the target site and achievement of the therapeutic concentration, leading to various effects associated with chemotherapy, including in terms of the functions of internal organs (liver, kidneys, heart), as well as infectious complications caused by impaired blood cell production by the bone marrow.^48^

**Figure 4:**
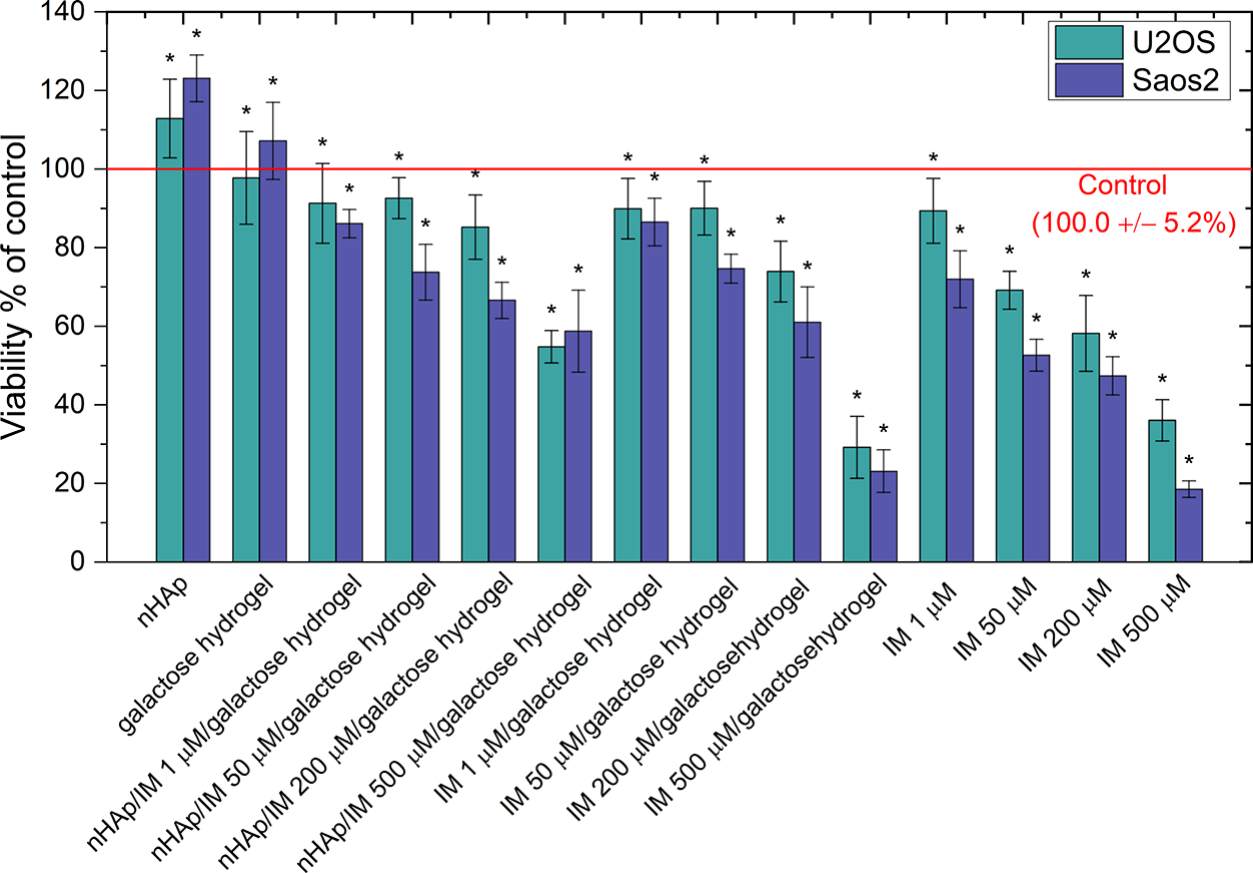
Evaluation of cell viability by colorimetric -MTT assay in U-2OS and Saos-2 cells incubated with a mixture of galactose hydrogel with hydroxyapatite and imatinib and a mixture of galactose hydrogel with the drug. Galactose hydrogel matrices and hydroxyapatite were also evaluated. Imatinib in concentrations adequate to those contained in the gels was used as a positive control; *p < 0.05, significant statistical differences compared to the negative control.

Targeted cancer treatment is the future of medicine. The hydrogels designed and described in this paper contain the commonly used cytostatic imatinib. Their purpose is to slower be sustained the release of the drug into the immediate focus of the cancer cells. The effect of the tested hydrogels on the viability of cell cultures was evaluated in the MTT assay: (1) nHAP/IM/galactose hydrogel of 1, 50, 200, and 500 μM; (2) IM/galactose hydrogel at the same concentrations. In parallel, a solution of IM at the same concentrations was applied directly to the cells as a positive control. nHAP/galactose hydrogel matrices were also tested. The hydrogels tested and the effect of imatinib on cell viability are summarized in Figure 4 (MTT assay). Neither nHAp nor galactose hydrogel affected cell metabolism and cell viability (assessment of morphology and mitochondrial activity by light microscopy and MTT assay, respectively). A concentration-dependent decrease in cell viability was observed in all gel matrices tested and for imatinib administered in solution. The nHAp/IM/galactose hydrogel did not significantly reduce cell culture viability compared to the imatinib administered alone. After treatment, a statistically significant reduction in Saos-2 cell viability was observed with all gels and imatinib solutions. For U-2OS cells, a statistically significant decrease was observed in the concentration range of 50–500 μM imatinib solution and 200–500 μM imatinib in the galactose hydrogel.

In contrast, when nHAp was added to the hydrogel matrix, a statistically significant decrease in U-2OS cell viability was observed only at the highest imatinib concentration. It is worth noting that the galactose gel with imatinib at a concentration of 500 μM shows a comparable cytotoxic effect against the Saos-2 cell line and a slightly stronger impact against U-2OS. It is important to remember that even the most effective anticancer drugs also cause strong toxicity towards normal cells and are associated with severe damage to host cells. In our earlier work, it was shown that hydrogels tested without the drug (imatinib) had no cytotoxic effect on normal fibroblast cells.^11^ It was also revealed that the action of nHAp alone with particles smaller than 40 nm increased the cytotoxic effect on hepatoma cancer cells.^49^ However, we did not observe such an effect in bone tumors. At the same time, importantly, they did not cause an increase in mitochondrial activity. Therefore, the increased cell viability of both lines after hydrogel treatment is not a cause for concern. When drugs are administered in the traditional form, they can induce multidrug resistance (MDR). Consequently, the drug is excreted from the tumor cells and the expected cytotoxic effect against tumor cells is then not achieved. Injecting the drug-hydrogel directly into the bone can result in a gradual release of the drug at the target site over a longer time, preventing the development of MDR and, consequently, the ineffectiveness of drug therapy.

**Figure 5:**
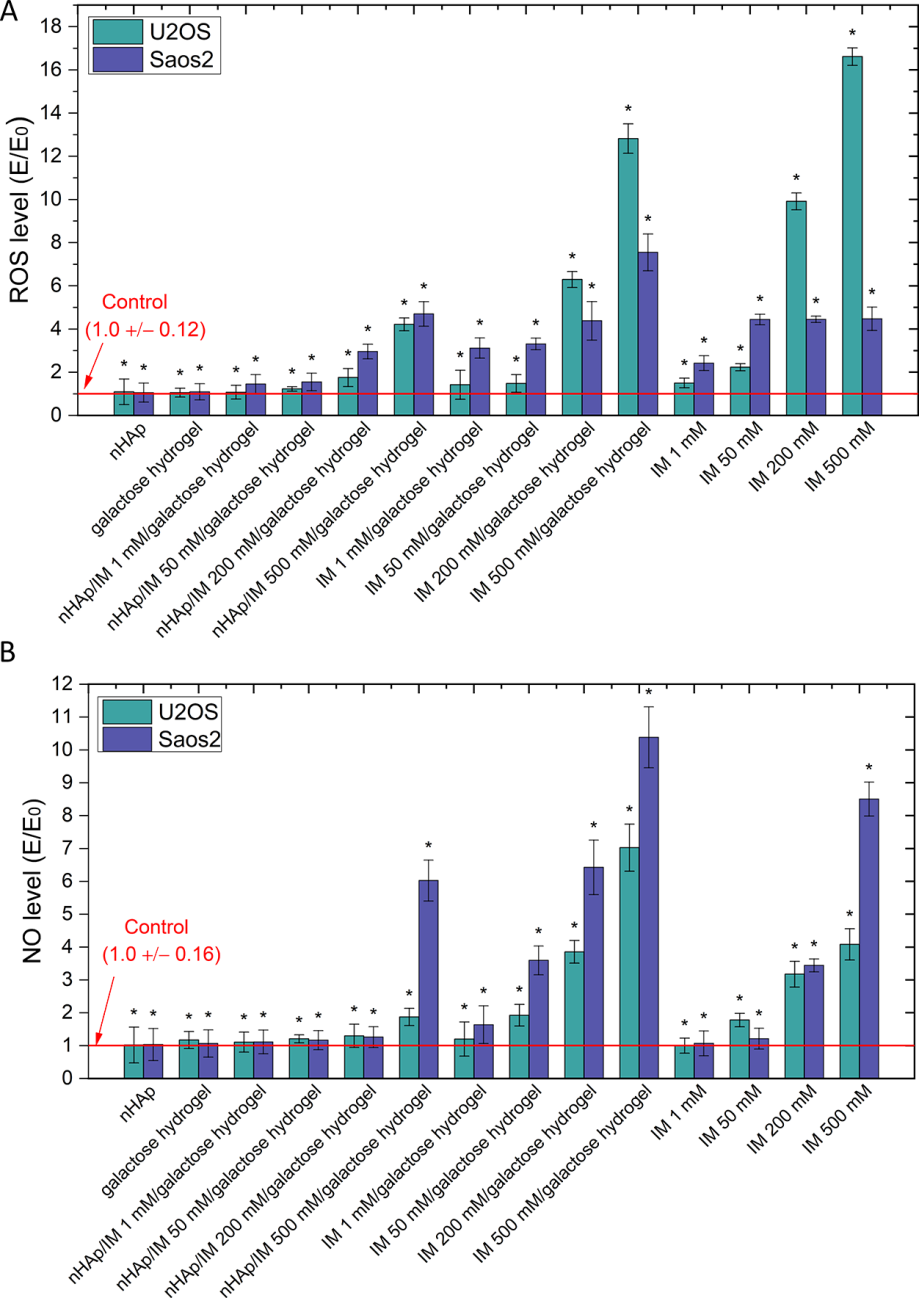
(A) Level of oxygen free radicals by the DCFDA assay; (B) level of nitrogen-free radical formation by the Griess reagent assay; performed on two cell lines from osteosarcoma patients (U-2OS and Saos-2); incubated with galactose hydrogel matrices and nHAp hydrogels; matrices containing the drug imatinib and the drug itself at different concentrations, which was also used as an additive to the matrices; * *p* < 0.05- significant statistical differences compared to negative control.

Mitochondrial activity decreases with increased reactive oxygen species (ROS) and nitrogen oxide (NO) levels. Based on the conducted research assessing the levels of radicals, diagrams were prepared to show the results in Figure 5A,B for the ROS and NO levels, respectively. Incubation with the nHAp/galactose hydrogels and galactose alone resulted in no increase in ROS or NO. However, the combination of nHAp with the galactose hydrogel and imatinib resulted in a statistically significant increase in the ROS level (at a concentration of 200 and 500 μM) and NO (500 μM) in both tested cell lines. On the other hand, imatinib only in the galactose hydrogel caused a much greater increase in ROS and NO levels in the entire range of imatinib concentrations tested in the Saos-2 cell line (excluding 1 μM for nitric oxide levels) and at concentrations of 200 and 500 μM in the U-2OS cell culture. In turn, imatinib in the form of direct contact with cell cultures increased the level of ROS in the entire range of tested concentrations in the Saos-2 cell line and in the concentration range of 50–500 μM in U-2OS cultures. On the other hand, the increase in the NO level was statistically significant at the concentrations of 200 and 500 μM in Saos-2 cultures and 50–500 μM in U-2OS cells. Interestingly, the increase in NO levels was greater after using the galactose hydrogel at concentrations of 50–500 μM compared to the same concentrations added directly to the cell cultures of both U-2OS and Saos-2.

**Scheme 1:**
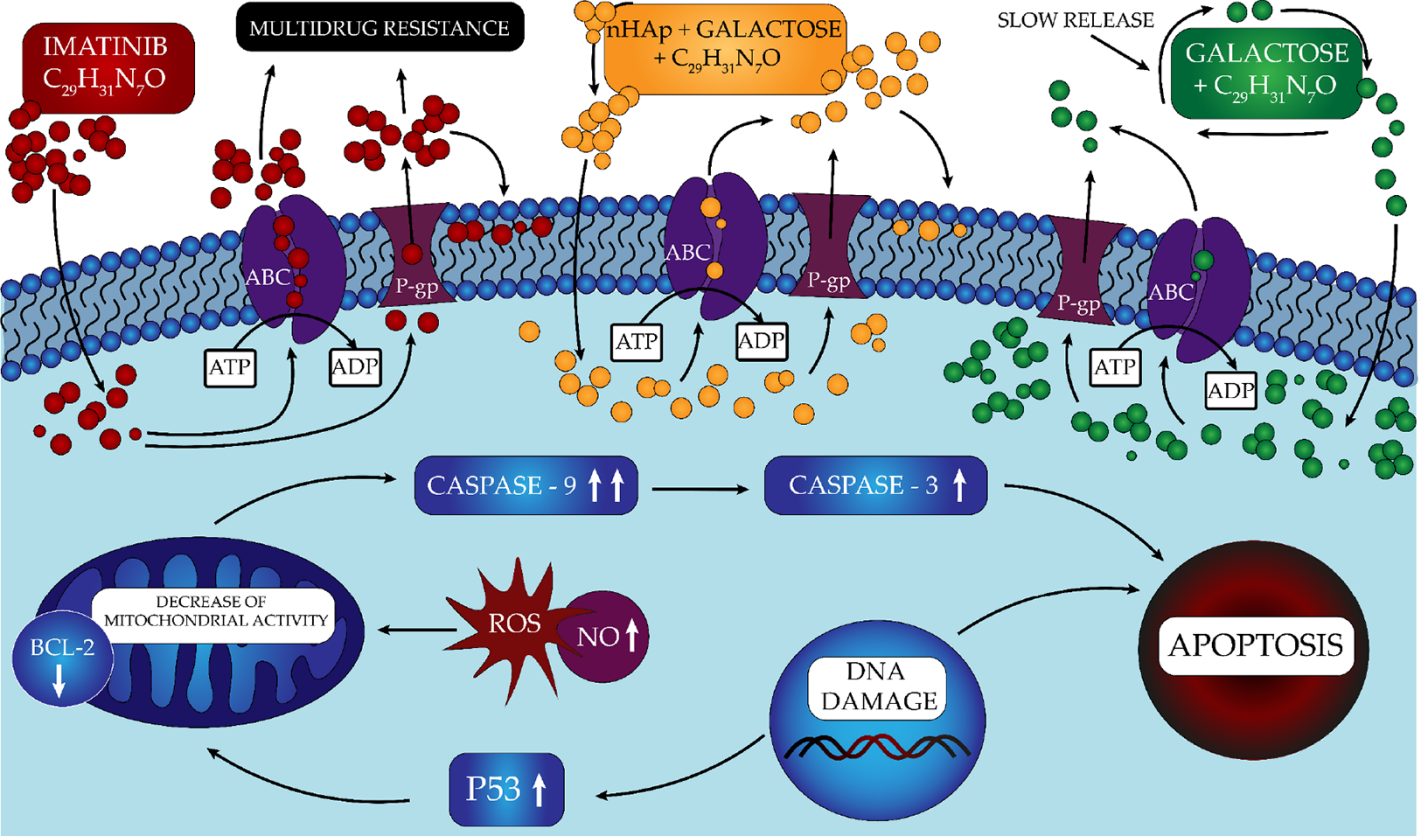
Potential Mechanism of Different Forms of Imatinib Administration in Saos-2 and U2OS cells (Direct and Two Different Hydrogel Patterns)

It is known that imatinib works by increasing the apoptosis of cancer cells. The effect of modifying the administration of imatinib, in the form of two types of hydrogels, on changes in the expression of signaling particles related to the apoptosis process was investigated (Scheme 1). The results (Table 1) showed a strong upregulation of both caspase 3 and caspase 9 expressions, as well as an increase in p53 and a slight decrease in BCL-2 expression. It is well known that imatinib inhibits BCR-Abl kinase by binding to the external domain. It then activates the MAP kinase pathway and, finally, DNA damage and apoptosis of cells. In osteosarcoma, many signaling targets are disrupted. For example, the expression of p21 and p27 proteins that enhance CDK expression is inhibited.

**Table 1:** Level of p53, BCL-2, Caspase 3 and Caspase 9, and Osteosarcoma Cells (U-2OS and Saos-2) Incubated with Different Forms of Hydrogels Containing the Drug at Various Concentrations and the Drug Imatinib Alone

	nHAp/IM/galactose hydrogel [μM]				IM/galactose hydrogel [μM]				imatinib (IM) [μM]			
signaling molecules	1	50	200	500	1	50	200	500	1	50	200	500
p53 [pg/mL]	2.52	3.44	4.49	5.67	3.42	4.46	5.53	7.63	3.40	4.42	5.62	7.52
BCL-2 [pg/mL]	0.60	0.64	0.56	0.55	0.46	0.45	0.45	0.43	0.49	0.47	0.41	0.41
caspase 3 [ng/mL]	0.24	0.31	0.39	0.52	0.40	0.60	0.84	1.23	0.35	0.46	0.71	0.95
caspase 9 [ng/mL]	3.18	5.71	7.20	9.89	3.87	6.59	9.15	11.66	3.54	5.91	7.76	8.27

Consequently, there is an uncontrolled increase in the amount of cell division. In patients with osteosarcoma, inhibition of the suppressor protein p53 is also observed. At the same time, an increase in BCL-2 activity is observed in this tumor—the p53 protein influences many intracellular processes, including activation of DNA repair and the induction of apoptosis. In the context of osteosarcoma, 10–39% inactivation of the p53 protein was found. Naturally, p53 expression is observed in the U2-OS line, while p53 expression is not shown in the Saos-2 line. Therefore, the signaling parameters were evaluated after incubation with the tested hydrogels and imatinib with the U-2OS line. As a result of the increased p53 protein, we can assume that there is an increase in DNA strand damage and a decrease in the level of BCL-2 because of an increase in apoptosis of cancer cells. Two pathways initiate the process of apoptosis: exogenous, dependent on death receptors, and internal, i.e., mitochondrial. Additionally, a pseudoreceptor pathway can be distinguished in which cell death is ultimately induced by granzyme B (GZMB) and granzyme A (GZMA). The external, internal and GZMB-dependent pathways initiating the apoptosis process converge in the executive phase, i.e., at the site of caspase-3 activation. In turn, GZMB starts apoptosis bypassing caspase 3.^50,51^ Based on the determined concentrations of caspase 3 and 9, it indicates that administration of IM in the hydrogel form induces apoptosis in the caspase-3 dependent pathway and in the pseudoreceptor pathway. In contrast, IM causes the activation of the caspase 3-dependent apoptosis process. On the other hand, in the case of the Saos-2 line, the increase in carcinogenic death will mainly result from the increase in the level of ROS and NO, which reduces mitochondrial activity and, consequently, cell death by apoptosis.

Moreover, while an increase in NO levels in p53-deficient Saos-2 cells decreased cell survival, it was seeded compared to U-2OS cells that have wild-type p53. Thus, the selective induction of the apoptotic pathway by NO levels may be a valuable adjunct to cancer chemotherapy by reducing the survival of p53-deficient cancer cells.

The study also determined the effect of imatinib and the form of its administration on the activity of *P*-glycoprotein. It was noticed that the administration of imatinib in the form of both galactose and nHAp hydrogels caused a significant increase in the accumulation of Rh-123 in cells (Table 2). Therefore, it is likely that the administration of imatinib in a slower-release hydrogel reduces the risk of developing multidrug resistance by inhibiting P_gp_.

**Table 2:** Relationship between Glycoprotein-P (P_gp_) Activity and Osteosarcoma Cells (U-2OS and Saos-2) Incubated with Different Forms of Hydrogels Containing the Drug at Various Concentrations and the Drug Imatinib Alone

			nHAp/IM/galactose hydrogel [μM]				IM/galactose hydrogel [μM]				imatinib (IM) [μM]			
cell lines	signaling molecules	control	1	50	200	500	1	50	200	500	1	50	200	500
U2OS	P_gp_	32,731	32,843	38,445	42,858	51,042	35,356	37,481	45,170	52,111	28,952	36,877	33,566	36,877
Saos-2	P_gp_	31,996	32,544	35,334	36,557	39,377	35,528	40,819	43,521	53,897	32,843	35,356	38,445	42,858

## 4 Conclusions

An investigation was conducted to determine how the rough surface of a hydroxyapatite-modified galactose hydrogel affects the release of amorphous imatinib and, consequently, its interaction with the studied osteosarcoma cells. It was found that the rough surface of galactose hydrogel has little effect on the drug release. The cytotoxic and pro-oxidative activity of the materials was slightly weaker than those administered directly. However, more p53 and BCL-2 proteins were observed in the hydrogel form compared to the direct administration. At the same time, it was observed that the concentrations of caspase 3 were at a similar level regardless of the form of administration—directly to the culture or hydrogels. In addition, compared to direct administration, an increase in the level of caspase-9 in both hydroxyapatite-loaded and unloaded hydrogels was observed, indicating the activation of the pseudoreceptor pathway and not only the caspase-3 dependent pathway. Furthermore, the administration of nHAp-loaded hydrogels significantly increased the accumulation of Rh-123 in the cells. Therefore, administrating a hydrogel to them is expected to reduce the risk of multidrug resistance. However, compared to other internal organs, the bones are poorly supplied with blood. At the same time, vascularization and multicellularity with extensive paracrine and intercellular interactions generate an environment highly conducive to the metastasis of tumor cells. Therefore, the standard oral form of cytostatic therapy shows little pharmacological efficacy, while the proposed new drug delivery is promising due to the direct surgical administration of the drug and a reduced risk of developing multidrug resistance.
